# Bis{(*E*)-3-[(2-hy­droxy­benzyl­idene)amino]­prop­yl}ammonium chloride

**DOI:** 10.1107/S1600536812045424

**Published:** 2012-11-10

**Authors:** Murad A. AlDamen, Salim F. Haddad

**Affiliations:** a Department of Chemistry, The University of Jordan, Amman 11942, Jordan

## Abstract

The title salt, C_20_H_26_N_3_O_2_^+^·Cl^−^, lies across a twofold crystallographic axis with the central N atom of the cation and the chloride anion sitting on this axis, *Z*′ = 0.5. There is an intra­molecular hydrogen bond between the hy­droxy H atom and the imino N atom. The chloride anion and the cation are connected into chains along the *a* axis by an N—H⋯Cl hydrogen bond. In the crystal, the chains are linked *via* C—H⋯Cl interactions forming two-dimensional networks lying parallel to (101).

## Related literature

For applications of similar ligands, see: Taha *et al.* (2011*a* ▶,*b* ▶). For analgous structures, see: Ramazani *et al.* (2006 ▶); Cheng *et al.* (2009 ▶); Chen *et al.* (2011 ▶); Pavel *et al.* (2007 ▶).
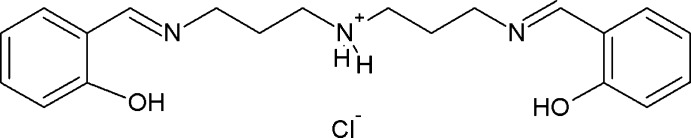


## Experimental

### 

#### Crystal data

C_20_H_26_N_3_O_2_^+^·Cl^−^
*M* *_r_* = 375.88
Orthorhombic, 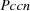

*a* = 4.9848 (3) Å
*b* = 38.714 (2) Å
*c* = 10.3299 (7) Å
*V* = 1993.5 (2) Å^3^
*Z* = 4
Mo *K*α radiation
μ = 0.21 mm^−1^
*T* = 100 K
0.52 × 0.37 × 0.04 mm

#### Data collection

Oxford Diffraction Xcalibur (Eos, Gemini ultra) diffractometer
Absorption correction: analytical [*CrysAlis PRO* (Oxford Diffraction, 2009 ▶), based on expressions derived from Clark & Reid (1995 ▶)] *T*_min_ = 0.94, *T*_max_ = 0.992
8874 measured reflections
2040 independent reflections
1654 reflections with *I* > 2σ(*I*)
*R*_int_ = 0.049

#### Refinement

*R*[*F* ^2^ > 2σ(*F* ^2^)] = 0.076
*wR*(*F* ^2^) = 0.150
*S* = 1.21
2040 reflections
119 parameters
Only H-atom displacement parameters refined
Δρ_max_ = 0.39 e Å^−3^
Δρ_min_ = −0.88 e Å^−3^

### 

Data collection: *CrysAlis PRO* (Oxford Diffraction, 2009 ▶); cell refinement: *CrysAlis PRO*; data reduction: *CrysAlis PRO*; program(s) used to solve structure: *SHELXS97* (Sheldrick, 2008 ▶); program(s) used to refine structure: *SHELXL97* (Sheldrick, 2008 ▶); molecular graphics: *OLEX2* (Dolomanov *et al.*, 2009 ▶); software used to prepare material for publication: *OLEX2*.

## Supplementary Material

Crystal structure: contains datablock(s) I, global. DOI: 10.1107/S1600536812045424/go2068sup1.cif

Structure factors: contains datablock(s) I. DOI: 10.1107/S1600536812045424/go2068Isup2.hkl

Supplementary material file. DOI: 10.1107/S1600536812045424/go2068Isup3.cml

Additional supplementary materials:  crystallographic information; 3D view; checkCIF report

## Figures and Tables

**Table 1:** Hydrogen-bond geometry (Å, °)

*D*—H⋯*A*	*D*—H	H⋯*A*	*D*⋯*A*	*D*—H⋯*A*
N1—H1*A*⋯Cl1	0.91	2.22	3.128 (2)	176
O1—H1⋯N2	0.97	1.70	2.577 (4)	148
C1—H1*C*⋯Cl1^i^	0.97	2.70	3.642 (4)	164

Symmetry code: (i) 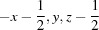.

**Fig. 1.**
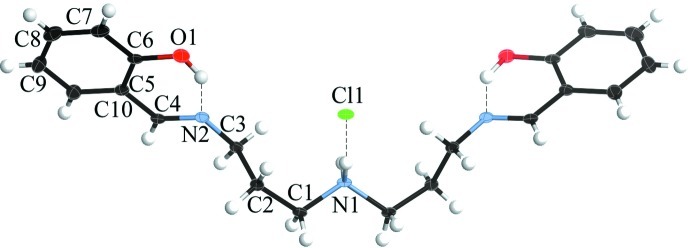
Asymmetric units and the atom-numbering schemes for (I) with thermal displacement ellipsoids at 30% probability (except hydrogens).

**Fig. 2.**
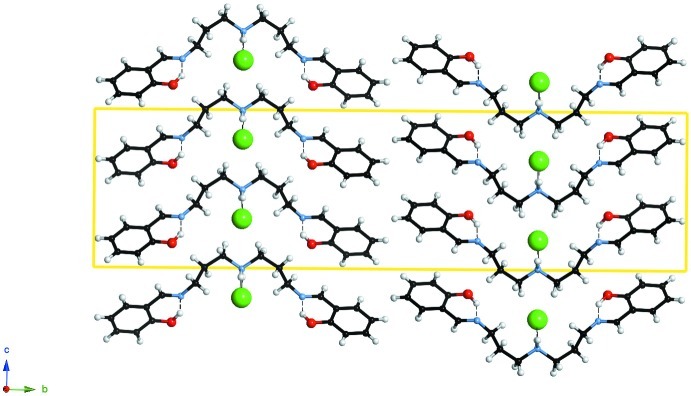
View of the molecular acking diagram, view perpendicular to the bc plane.

## supplementary crystallographic information

### Comment

Tetradentate Schiff bases are flexible ligands and difficult to crystallize and most of the crystal structures published for these ligands are complexes with transition metals or anthanides/actinides.

As far as we can determine there are no published structures for this type of ligand and published data for these types of ligand with antimicrobial or of interst because of their optical properies have been were has been obtained from solid state techniques like NMR or elemental analysis Taha *et al.* (2011*a*); Taha *et al.* (2011*b*). We crystallized (I) as salt. (Fig. 1). The molecule sits across a two-fold crystallographic axis with the ammonium nitrogen atom, N1, and the Cl anion sitting on the two-fold axis.

The bond lengths of N1—C1 and N2—C3 are 1.501 (4) and 1.456 (4) respectively indicate that these are single bonds and the bond length of N2—C4 (1.273 (4) Å) shows that this is a double bond exhibits a double bond. The distances are in agreement with other salicylic and dipropylamino ligands and complexes found in literature (Ramazani *et al.* (2006), Cheng *et al.* (2009), Pavel *et al.* (2007) & Chen *et al.* (2011)).

There is an intramolecular hydrogen bond between the phenolic oxygen, O1 and N2, Table 1, Fig1 The N1—HH1A···Cl1 hydrogen bond connects the chloride ion to two adjacent ligands to form chains which run parallel to the *a* axis, Table 1 and Fig. 1. In addition there is a short contact between C1 and Cl1, Table 1. There are no C–H···π interactions nor is there any π-π stacking.

### Experimental

Facile condensation of 3,3 –Diaminodipropylamine and salicylaldehyde 1:2 molar ratio afforded a neutral condensate as following:

3,3 –Diaminodipropylamine (30 ml, 0.208 mol) was added dropwise to a solution of salicylaldehyde (50.7 g, 0.416 mol) in absolute ethanol (250 ml); the solution became instantly yellow. The mixture was heated for 1 h at 50°C. Evaporation of the solvent under reduced pressure afforded the desired compound in 95% yield as yellow oil. The obtained Schiff base was then mixed with cyanuric acid in ethanol with few drops of 1*M* HCl added. The mixture then stir heated (40°C) for 24 then filtered. After few days crystals suitable for X-ray diffraction were formed.

### Refinement

H atoms were treated as riding atoms with C—H(aromatic), 0.93Å and C—H(CH_2_), 0.97Å, with *U*_iso_ = 1.2Ueq(C). The hydrogens attached N1 and O1 were located on a difference map and refined as riding atoms with *U*_iso_ = 1.2Ueq(N1) and *U*_iso_ = 1.5Ueq(O1). The positions of these latter atoms was confirmed on a final difference map. The position of the Cl anion was chosen so that it formed a hydrogen bonded asymmetric unit.

### Crystal data

**Table:**

C_20_H_26_N_3_O_2_^+^·Cl^−^	*D*_x_ = 1.252 Mg m^−^^3^
*M**_r_* = 375.88	Mo *K*α radiation, λ = 0.7107 Å
Orthorhombic, *P**c**c**n*	Cell parameters from 2556 reflections
*a* = 4.9848 (3) Å	θ = 3.7–26.3°
*b* = 38.714 (2) Å	µ = 0.21 mm^−^^1^
*c* = 10.3299 (7) Å	*T* = 100 K
*V* = 1993.5 (2) Å^3^	Prism, white
*Z* = 4	0.52 × 0.37 × 0.04 mm
*F*(000) = 800	

### Data collection

**Table:**

Oxford Diffraction Xcalibur (Eos, Gemini ultra) diffractometer	2040 independent reflections
Radiation source: fine-focus sealed tube	1654 reflections with *I* > 2σ(*I*)
Graphite monochromator	*R*_int_ = 0.049
Detector resolution: 16.0122 pixels mm^-1^	θ_max_ = 26.4°, θ_min_ = 4.0°
ω scans	*h* = −6→5
Absorption correction: analytical [*CrysAlis PRO* (Oxford Diffraction, 2009), based on expressions derived from Clark & Reid (1995)]	*k* = −48→48
*T*_min_ = 0.94, *T*_max_ = 0.992	*l* = −12→12
8874 measured reflections	

### Refinement

**Table:**

Refinement on *F*^2^	Primary atom site location: structure-invariant direct methods
Least-squares matrix: full	Secondary atom site location: difference Fourier map
*R*[*F*^2^ > 2σ(*F*^2^)] = 0.076	Hydrogen site location: inferred from neighbouring sites
*wR*(*F*^2^) = 0.150	Only H-atom displacement parameters refined
*S* = 1.21	*w* = 1/[σ^2^(*F*_o_^2^) + (0.0141*P*)^2^ + 6.1394*P*] where *P* = (*F*_o_^2^ + 2*F*_c_^2^)/3
2040 reflections	(Δ/σ)_max_ < 0.001
119 parameters	Δρ_max_ = 0.39 e Å^−^^3^
0 restraints	Δρ_min_ = −0.88 e Å^−^^3^

### Special details

**Table:**

Geometry. All esds (except the esd in the dihedral angle between two l.s. planes) are estimated using the full covariance matrix. The cell esds are taken into account individually in the estimation of esds in distances, angles and torsion angles; correlations between esds in cell parameters are only used when they are defined by crystal symmetry. An approximate (isotropic) treatment of cell esds is used for estimating esds involving l.s. planes.

### Fractional atomic coordinates and isotropic or equivalent isotropic displacement parameters (Å^2^)

**Table:**

	*x*	*y*	*z*	*U*_iso_*/*U*_eq_	
O1	−0.1873 (5)	0.13196 (7)	0.8454 (3)	0.0340 (7)	
H1	−0.0740	0.1460	0.7900	0.051*	
N1	0.2500	0.2500	0.5049 (4)	0.0174 (8)	
H1A	0.1010	0.2508	0.5560	0.021*	
N2	0.1804 (6)	0.14754 (7)	0.6799 (3)	0.0229 (7)	
C1	0.2328 (7)	0.21793 (8)	0.4236 (3)	0.0213 (7)	
H1B	0.4094	0.2126	0.3897	0.026*	
H1C	0.1150	0.2223	0.3507	0.026*	
C2	0.1289 (7)	0.18696 (8)	0.4984 (3)	0.0230 (8)	
H2A	0.0861	0.1688	0.4373	0.028*	
H2B	−0.0364	0.1935	0.5414	0.028*	
C3	0.3212 (7)	0.17249 (9)	0.5993 (3)	0.0236 (8)	
H3A	0.4716	0.1614	0.5566	0.028*	
H3B	0.3901	0.1911	0.6526	0.028*	
C4	0.2480 (7)	0.11586 (8)	0.6762 (3)	0.0226 (7)	
H4	0.3885	0.1092	0.6225	0.027*	
C5	0.1112 (7)	0.08970 (9)	0.7539 (3)	0.0234 (8)	
C6	−0.1012 (7)	0.09900 (9)	0.8369 (3)	0.0257 (8)	
C7	−0.2244 (9)	0.07364 (10)	0.9119 (4)	0.0345 (9)	
H7	−0.3635	0.0796	0.9676	0.041*	
C8	−0.1417 (9)	0.03980 (11)	0.9042 (4)	0.0388 (10)	
H8	−0.2260	0.0231	0.9547	0.047*	
C9	0.0656 (9)	0.03018 (10)	0.8224 (4)	0.0394 (10)	
H9	0.1205	0.0073	0.8177	0.047*	
C10	0.1893 (8)	0.05520 (9)	0.7478 (4)	0.0325 (9)	
H10	0.3278	0.0489	0.6924	0.039*	
Cl1	−0.2500	0.2500	0.68790 (11)	0.0223 (3)	

### Atomic displacement parameters (Å^2^)

**Table:**

	*U*^11^	*U*^22^	*U*^33^	*U*^12^	*U*^13^	*U*^23^
O1	0.0353 (16)	0.0380 (14)	0.0286 (15)	0.0007 (12)	0.0106 (13)	−0.0020 (12)
N1	0.0110 (18)	0.0282 (19)	0.0131 (19)	−0.0011 (17)	0.000	0.000
N2	0.0248 (16)	0.0259 (14)	0.0180 (15)	−0.0025 (12)	−0.0038 (13)	−0.0005 (12)
C1	0.0195 (16)	0.0289 (17)	0.0156 (16)	−0.0015 (15)	−0.0012 (14)	−0.0030 (13)
C2	0.0200 (18)	0.0259 (17)	0.0230 (19)	−0.0021 (14)	−0.0039 (15)	−0.0010 (14)
C3	0.0235 (19)	0.0263 (17)	0.0208 (18)	−0.0040 (14)	−0.0011 (15)	−0.0029 (14)
C4	0.0210 (17)	0.0310 (17)	0.0158 (16)	0.0000 (15)	0.0007 (15)	−0.0017 (14)
C5	0.0202 (18)	0.0298 (18)	0.0201 (18)	−0.0024 (14)	−0.0037 (15)	0.0004 (14)
C6	0.0252 (19)	0.0342 (19)	0.0178 (18)	−0.0038 (16)	−0.0034 (15)	−0.0008 (15)
C7	0.031 (2)	0.048 (2)	0.024 (2)	−0.0092 (19)	0.0037 (18)	0.0034 (17)
C8	0.037 (2)	0.045 (2)	0.035 (2)	−0.0136 (19)	−0.0037 (19)	0.0133 (19)
C9	0.045 (3)	0.030 (2)	0.043 (3)	0.0001 (18)	0.000 (2)	0.0099 (18)
C10	0.032 (2)	0.0319 (19)	0.033 (2)	0.0000 (17)	0.0020 (18)	0.0014 (17)
Cl1	0.0132 (5)	0.0368 (6)	0.0169 (5)	−0.0037 (5)	0.000	0.000

### Geometric parameters (Å, º)

**Table:**

O1—C6	1.349 (4)	C3—H3B	0.9700
O1—H1	0.9705	C4—C5	1.461 (5)
N1—C1^i^	1.501 (4)	C4—H4	0.9300
N1—C1	1.501 (4)	C5—C10	1.393 (5)
N1—H1A	0.9117	C5—C6	1.409 (5)
N2—C4	1.272 (4)	C6—C7	1.394 (5)
N2—C3	1.456 (4)	C7—C8	1.376 (6)
C1—C2	1.518 (4)	C7—H7	0.9300
C1—H1B	0.9700	C8—C9	1.386 (6)
C1—H1C	0.9700	C8—H8	0.9300
C2—C3	1.522 (5)	C9—C10	1.383 (5)
C2—H2A	0.9700	C9—H9	0.9300
C2—H2B	0.9700	C10—H10	0.9300
C3—H3A	0.9700		
			
C6—O1—H1	107.8	H3A—C3—H3B	108.2
C1^i^—N1—C1	112.0 (3)	N2—C4—C5	121.8 (3)
C1^i^—N1—H1A	110.0	N2—C4—H4	119.1
C1—N1—H1A	107.8	C5—C4—H4	119.1
C4—N2—C3	119.6 (3)	C10—C5—C6	118.8 (3)
N1—C1—C2	112.8 (3)	C10—C5—C4	120.6 (3)
N1—C1—H1B	109.0	C6—C5—C4	120.5 (3)
C2—C1—H1B	109.0	O1—C6—C7	119.3 (3)
N1—C1—H1C	109.0	O1—C6—C5	121.3 (3)
C2—C1—H1C	109.0	C7—C6—C5	119.3 (3)
H1B—C1—H1C	107.8	C8—C7—C6	120.4 (4)
C1—C2—C3	115.1 (3)	C8—C7—H7	119.8
C1—C2—H2A	108.5	C6—C7—H7	119.8
C3—C2—H2A	108.5	C7—C8—C9	121.0 (4)
C1—C2—H2B	108.5	C7—C8—H8	119.5
C3—C2—H2B	108.5	C9—C8—H8	119.5
H2A—C2—H2B	107.5	C10—C9—C8	119.0 (4)
N2—C3—C2	109.4 (3)	C10—C9—H9	120.5
N2—C3—H3A	109.8	C8—C9—H9	120.5
C2—C3—H3A	109.8	C9—C10—C5	121.5 (4)
N2—C3—H3B	109.8	C9—C10—H10	119.3
C2—C3—H3B	109.8	C5—C10—H10	119.3
			
C1^i^—N1—C1—C2	−161.9 (3)	C10—C5—C6—C7	1.0 (5)
N1—C1—C2—C3	−70.5 (4)	C4—C5—C6—C7	−178.7 (3)
C4—N2—C3—C2	114.2 (3)	O1—C6—C7—C8	179.5 (4)
C1—C2—C3—N2	169.3 (3)	C5—C6—C7—C8	−0.6 (6)
C3—N2—C4—C5	−179.2 (3)	C6—C7—C8—C9	0.2 (6)
N2—C4—C5—C10	180.0 (3)	C7—C8—C9—C10	0.0 (6)
N2—C4—C5—C6	−0.4 (5)	C8—C9—C10—C5	0.4 (6)
C10—C5—C6—O1	−179.2 (3)	C6—C5—C10—C9	−0.8 (6)
C4—C5—C6—O1	1.2 (5)	C4—C5—C10—C9	178.8 (4)

Symmetry code: (i) −*x*+1/2, −*y*+1/2, *z*.

### Hydrogen-bond geometry (Å, º)

**Table:**

*D*—H···*A*	*D*—H	H···*A*	*D*···*A*	*D*—H···*A*
N1—H1*A*···Cl1	0.91	2.22	3.128 (2)	176
O1—H1···N2	0.97	1.70	2.577 (4)	148
C1—H1*C*···Cl1^ii^	0.97	2.70	3.642 (4)	164

Symmetry code: (ii) −*x*−1/2, *y*, *z*−1/2.
